# Brain fingerprint and subjective mood state across the menstrual cycle

**DOI:** 10.3389/fnins.2024.1432218

**Published:** 2024-12-06

**Authors:** Lorenzo Cipriano, Marianna Liparoti, Emahnuel Troisi Lopez, Antonella Romano, Laura Sarno, Camille Mazzara, Fabio Alivernini, Fabio Lucidi, Giuseppe Sorrentino, Pierpaolo Sorrentino

**Affiliations:** ^1^Department of Motor Sciences and Wellness, University of Naples “Parthenope”, Naples, Italy; ^2^Department of Philosophical, Pedagogical and Quantitative-Economic Sciences, University of Chieti-Pescara "G. d'Annunzio", Chieti, Italy; ^3^Institute of Applied Sciences and Intelligent Systems of National Research Council, Pozzuoli, Italy; ^4^Department of Neurosciences, Reproductive Science and Dentistry, University of Naples “Federico II”, Naples, Italy; ^5^Department of Promoting Health, Maternal-Infant and Specialized Medicine “G. D’Alessandro”, University of Palermo, Palermo, Italy; ^6^Institute of Biophysics of National Research Council, Palermo, Italy; ^7^Department of Social and Developmental Psychology, Sapienza University of Rome, Rome, Italy; ^8^ICS Maugeri Hermitage Napoli, via Miano, Naples, Italy; ^9^Institut de Neurosciences des Systèmes, Aix-Marseille Université, Marseille, France; ^10^Department of Biomedical Sciences, University of Sassari, Sassari, Italy

**Keywords:** brain fingerprint, brain connectivity, menstrual cycle, depression, self-esteem, well-being

## Abstract

**Background:**

Brain connectome fingerprinting represents a recent and valid approach in assessing individual identifiability on the basis of the subject-specific brain functional connectome. Although this methodology has been tested and validated in several neurological diseases, its performance, reliability and reproducibility in healthy individuals has been poorly investigated. In particular, the impact of the changes in brain connectivity, induced by the different phases of the menstrual cycle (MC), on the reliability of this approach remains unexplored. Furthermore, although the modifications of the psychological condition of women during the MC are widely documented, the possible link with the changes of brain connectivity has been poorly investigated.

**Methods:**

We conducted the Clinical Connectome Fingerprint (CCF) analysis on source-reconstructed magnetoencephalography signals in a cohort of 24 women across the MC.

**Results:**

All the parameters of identifiability did not differ according to the MC phases. The peri-ovulatory and mid-luteal phases showed a less stable, more variable over time, brain connectome compared to the early follicular phase. This difference in brain connectome stability in the alpha band significantly predicted the self-esteem level (*p*-value <0.01), mood (*p*-value <0.01) and five (environmental mastery, personal growth, positive relations with others, purpose in life, and self-acceptance) of the six dimensions of well-being (*p*-value <0.01, save autonomy).

**Conclusion:**

These results confirm the high reliability of the CCF as well as its independence from the MC phases. At the same time the study provides insights on changes of the brain connectome in the different phases of the MC and their possible role in affecting women’s subjective mood state across the MC. Finally, these changes in the alpha band share a predictive power on self-esteem, mood and well-being.

## 1 Introduction

Recent years have seen the growing interest in searching for the subject-specific network stability with the purpose of reaching an individual identifiability on the basis of the brain functional connectome (Mallaroni et al., 2024; Finn et al., 2015; Van De Ville et al., 2021; Colenbier et al., 2023). From here arises the notion of brain fingerprinting, an approach utilized to define the connectome subject-specific characteristics (Finn et al., 2015; Amico and Goñi, 2018; Sareen et al., 2021). This methodology has been tested in several neurological diseases, revealing that individuals affected by neurodegenerative diseases showed reduced identifiability with respect to healthy subjects (Sorrentino et al., 2021; Svaldi et al., 2021). Interestingly, the reduced identifiability was able to predict individual motor, cognitive or behavioral features, giving rise to the concept of the Clinical Connectome Fingerprint (CCF; Sorrentino et al., 2021; Svaldi et al., 2021; Romano et al., 2022; Cipriano et al., 2023a; Troisi Lopez et al., 2023).

However, although the impact of different neurological conditions on CCF has been demonstrated, whether brain fingerprint is reliable and applicable, regardless of the cyclic physiological changes that take place in biological organisms on time scales ranging from a few seconds to weeks or months (Paranjpe and Sharma, 2005) remains poorly explored. Very recently, the stability of brain fingerprint was evaluated in a population of healthy young adults over a 24 h period, revealing that the functional connectome (FC) was stable during the daytime hours (between the morning and evening recordings), but after a night of sleep deprivation, the intra- and inter-identifiability was reduced. Consistently, the reduced stability of brain fingerprint following sleep deprivation was found to be negatively correlated with the effort perceived by participants in completing a cognitive task (Ambrosanio et al., 2024).

In consideration of the role played in the reproductive process, the menstrual cycle (MC) is one of the most important biological phenomena that takes place on a cyclical scale. However, the MC affects the woman’s life far beyond the reproductive function, inducing changes in the functioning of the central nervous system and in the women’s self-perception and behavioral states, as recently remarked by a very large study (3.3 million women across 109 different countries; Pierson et al., 2021). Furthermore, a large number of women suffer from MC related psychological changes ranging from not clinically relevant cycle-related emotional symptoms to overt premenstrual syndrome (PMS). Moreover, approximately 5–8% of women of reproductive age suffer from premenstrual dysphoric disorders (PMDD; Payne et al., 2009; Parker and Brotchie, 2010). PMDD is characterized by cyclic, debilitating cognitive, somatic and affective symptoms (depressed mood, irritability, affective lability, anxiety) which occur during the luteal phase, and greatly affect quality of life (Wittchen et al., 2002). In particular, self-esteem fluctuations have been related to the MC phases since the 90s (Bloch et al., 1997; Taylor, 1999), especially in the context of premenstrual syndrome. More recently, Brock et al. investigated whether self-esteem fluctuates over the MC, demonstrating that at premenstrual phase, negative self-esteem, anxiety and depression were higher whereas positive self-esteem was lower than at mid-cycle phase (Brock et al., 2016). The same is true for anxiety and depression that were found exacerbated in the few days before and during the menstrual period (Clayton, 2008). Most of these symptoms can occur in all the phases of the MC with a high prevalence in the late luteal phase when both estradiol and progesterone fall right before the menses (Le et al., 2020). This phase is commonly characterized by a poorer performance in emotion-related cognition (Le et al., 2020; Handy et al., 2022), a potential vulnerability to anxiety (Armbruster et al., 2014; Welz et al., 2016; Shayani et al., 2020) and minimal difficulties in executive functions (Solis-Ortiz et al., 2004; Dan et al., 2019). Additionally, the hormonal fall during this phase is also associated with important physical symptoms such as mastalgia, edema, headache, skin changes, muscle and joint pain as well as depression, anxiety insomnia and irritability (Modzelewski et al., 2024). These cyclic psychological, behavioral, and somatic symptoms get worse during the late luteal phase and improve after the onset of menses. This suggests that fluctuating ovarian hormones play a role in this mechanism (Yen et al., 2019). Progesterone (stable during most of the mid-luteal phase) decline prior to menses and higher concentrations in women blood have been associated with lower irritability and symptoms of fatigue in healthy women (Ziomkiewicz et al., 2012). Additionally, the estrogen receptor alpha gene polymorphism is associated with the risk of PMDD (Huo et al., 2007). However, to date, all these studies examining the MC and its relationship with cognition and mood in healthy woman have been unable to show consistent associations between these aspects and the different phases of the MC.

To date, the relationship between the MC-related changes in brain structure and function and women’s subjective mood state across the MC remains elusive (Dubol et al., 2021). However, recently, some structural and functional studies have reported the association between some specific brain features and subjective mood aspects. DTI studies, for example, have reported a greater white matter volume in the right uncinate fasciculus in patients with PMDD (as compared to controls) and demonstrated a positive correlation between premenstrual symptom severity and fractional anisotropy in the right superior longitudinal fasciculus (Gu et al., 2022). Studies on the brain metabolism (through PET scans) have emphasized the role played by the altered availability of the serotonin transporter during the luteal phase and the following temporary depletion of synaptic serotonin as a possible explanation of the mood changes observed during the luteal phase, supporting the clinical evidence of SSRIs efficacy in PMDD (Reilly et al., 2023; Sacher et al., 2023). Interestingly, a recent interventional study by Derntl et al. reported that the administration of estradiol valerate resulted in changes in effective connectivity in emotional-related networks, highlighting the potential utility of hormonal administration in the treatment of MC-related mental disorders that show a dysregulation of emotions (Derntl et al., 2024).

Taken together, the paucity of the exhaustive studies on this topic, the great social and economic impact (considering that several previous studies report that the prevalence of at least one premenstrual symptom can reach up to 90%; Dennerstein et al., 2012) as well as the broad interest in studying the relationship between the brain and behavior, mood and self-related aspects during the MC, suggest that there is more to be done in this field.

The aim of our work was to verify whether the brain fingerprint is stable during MC, thus establishing whether this technique is reliable and can be applied regardless of the MC phases. A further objective was to explore the functional connectome (FC) features and the contribution of specific brain areas to brain fingerprinting along MC. Finally, the possible relationship between changes of FC and the subjective mood state, assessed as subjective wellbeing and self-esteem was examined.

**Figure 1:**
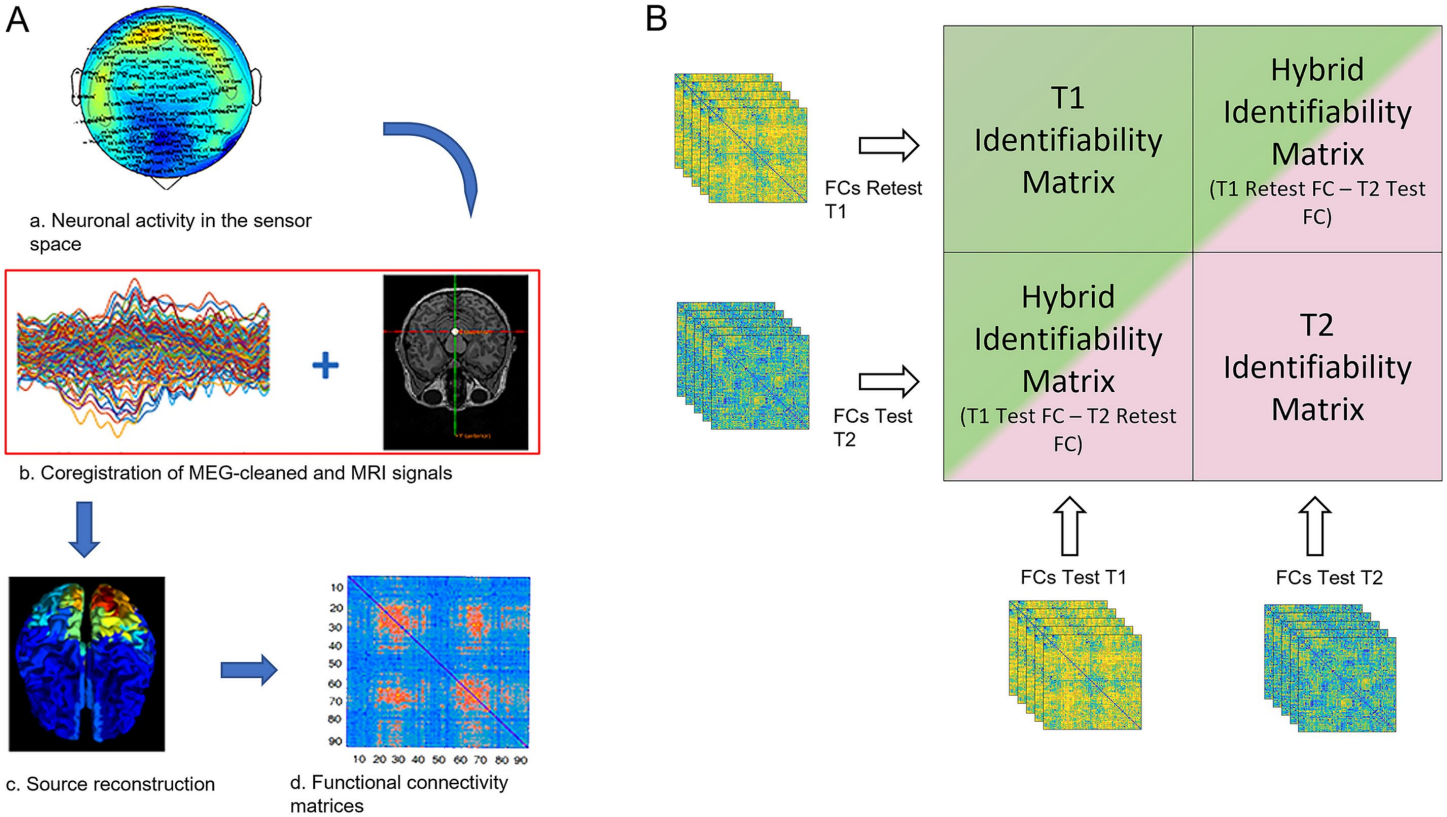
FCs processing and fingerprint analysis. (A) a: the neuronal activity was recorded using a 154-sensors magnetoencephalography (MEG); b: MEG signals were cleaned by removing noise and artifacts, coregistered with the subject-specific MRI scan for source reconstruction (c); d: functional connectivity matrix estimation based on the PLM. (B) The green and the pink blocks represent the two identifiability matrices of women in two different MC phases (in this explanatory figure: the T1 and T2 phases), resulting from the correlation of the test and re-test of the individual functional connectomes, in each phase (defined according to the MC phase) separately. Hybrid identifiability matrices (IMs) were created by crossing the FCs test of the T1 phase with the FCs retest of the T2 phase and *vice-versa*. The hybrid IMs contain the “cycle-specific fingerprinting” score (I-cyclical, IC) of each individual.

To scrutinize these aspects, we performed for each recruited woman a brain fingerprinting based on her whole functional connectome (Finn et al., 2015; Amico and Goñi, 2018; Sareen et al., 2021). More specifically, we used source-reconstructed magnetoencephalography (MEG) signals in a cohort of 24 healthy women without menstrual cycle dysfunction, premenstrual symptoms, anxiety and/or depression. We performed two separate recordings for each subject in three phases (early follicular, peri-ovulatory and mid-luteal) of the MC. After filtering the source-reconstructed signals in the canonical frequency bands, we used the phase linearity measurement (PLM; Baselice et al., 2019) to estimate the synchronization between regions, obtaining frequency-specific connectomes. Then, we estimated the identifiability rate of the women at each time point, based on the Pearson’s correlations between the connectomes. Furthermore, we compared the similarity between each subject’s connectome at a time point with the phases’ connectomes at the other time point, thereby obtaining a cycle-specific fingerprinting score of identifiability (I-cyclical, IC; Figure 1) that suggests how much the FC of each woman changed across the MC. To test the hypothesis that the IC score was predictive of the psychological subjective condition, we designed a multilinear regression model from the IC score of each subject. Lastly, we studied the nodal strength of each region of interest (ROI), thus defining the regions with the greatest contribution in predicting subjective features.

## 2 Materials and methods

### 2.1 Participants and experimental protocol

Twenty-four right-handed, native Italian speakers, heterosexual women with regular MC (mean cycle length 28.4 ± 1.3 days) were recruited. Exclusion criteria were: (1) use of hormonal contraceptives (or other hormone regulating medicaments) during the last 6 months before the recording (the use of these during the life was not an exclusion criteria); (2) pregnancy in the last 12 months; (3) chronic use of drugs able to affect the central nervous system; (4) alcohol, tobacco, and/ or coffee consumption 48 h prior to the MEG recordings; (5) absence of history of neuropsychiatric diseases and premenstrual dysphoric/depressive symptoms. Mood and/or anxiety symptoms were investigated by means of the Beck Depression Inventory (BDI; Beck et al., 1996) and Beck Anxiety Inventory (BAI; Beck and Steer, 1990), using a cut-off below 10 and 21, respectively, to exclude women with clinically evident depression and/or anxiety. To control for the influence of circadian rhythm, the time of testing varied no more than 2 h among testing sessions. To reduce the impact of sleep deprivation on brain connectivity, we recommended at least 8 h of sleep; the women did not report relevant sleep disturbances in the 24 h prior to the MEG acquisition. Additionally, none of the women were mothers at the time of the MEG acquisition. The intensity of the symptoms before and/or during menstruation was not specifically assessed. The subjects’ characteristics are shown in Table 1.

**Table 1:** Demographic and sex hormone data.

Demographic features	Mean (SD)					
Age (y)	26.29 (5.10, 22–40)					
Education (y)	16.54 (2.01)					
Menstrual cycle duration (days)	28.4 (1.5)					
Sex hormone blood levels	Early-follicular (T1)	Peri-ovulatory (T2)	Mid-luteal (T3)	T1vsT2, *p value*	T1vsT3, *p value*	T2vsT3, *p value*
LH (mIU/ml)	5.38 (2.34)	15.91 (11.88)	5.95 (4.04)	<0.001	ns	<0.001
FSH (mIU/ml)	7.28 (1.39)	7.65 (2.99)	3.88 (1.16)	ns	<0.001	<0.001
Progesterone (ng/ml)	0.30 (0.06)	1.12 (0.81)	5.75 (2.61)	ns	<0.001	<0.001
Estradiol (pg/ml)	33.94 (12.09)	134.27 (70.62)	97.42 (39.72)	<0.001	<0.001	<0.05
Affective/emotional tests	Early-follicular (T1)	Peri-ovulatory (T2)	Mid-luteal (T3)	T1vsT2, *p value*	T1vsT3, *p value*	T2vsT3, *p value*
BAI	7.62 (7.15)	7.88 (8.11)	5.92 (4.70)	ns	ns	ns
Rosenberg	22.58 (4.84)	23.08 (4.84)	23.83 (4.15)	ns	ns	ns
BDI	3.71 (3.47)	3.04 (3.16)	2.88 (2.82)	ns	ns	ns
Wellbeing	401.88 (50.84)	400.50 (51.73)	399.00 (45.40)	ns	ns	ns
Number of patients with first MEG acquisition in each time point	13 (T1)	3 (T2)	8 (T3)			

BAI, Beck Anxiety Index; BDI, Beck Depression Inventory; FSH, follicle stimulating hormone; LH, luteinizing hormone. Analysis of variance (ANOVA) of sex hormones according to the MC phases; the *p* values of *post-hoc* analysis between the MC phases are shown in the last three columns. ns, not significant.

All women were tested in three different time points of the MC, that is, in the early follicular phase (cycle day 1–4, low estradiol and progesterone, T1), during the peri-ovulatory phase (cycle day 13–15, high estradiol and low progesterone, T2) and in the mid-luteal phase (cycle day 21–23, high estradiol and progesterone, T3).

At each of the three time points along the cycle, all subjects underwent the following examinations: MEG recording, blood sampling for the hormone assay, and psychological evaluation (self-esteem, well-being, anxiety and depression). All participants underwent a transvaginal pelvic ultrasonography during the early follicular phase and a structural magnetic resonance imaging (MRI) after the last MEG recording. Hormone assays, MRI and ultrasound examination have been performed according to Liparoti et al. (2021).

### 2.2 Hormone assays

Each participant underwent venous blood sampling during each phase of the MC. The blood sampling was preceded from a 12 h of fast. Blood samples were collected in S-Monovette tubes (Sarstedt), containing gel with a clotting activator, according to predetermined standard operating procedure (Tuck et al., 2009). Samples were centrifuged at 4,000 rpm for 10 min, with a following serum collection, aliquot in 1.5 mL tubes (Sarstedt), and storage at −80°C until the analysis. Determination of estradiol followed different ranges according to the MC phase (range: 19.5–144.2 pg./mL in the early follicular phase; 63.9–356.7 pg./mL in the peri-ovulatory phase and 55.8–214.2 pg./mL in the mid-luteal phase) with the detection limit of 11.8 pg./mL. Progesterone ranges were ND–1.4 ng/mL in the early follicular phase, ND–2.5 ng/mL in the peri-ovulatory phase and 2.5–28.03 ng/mL in the mid-luteal phase with a detection limit of 0.2 ng/mL. LH ranges were 1.9–12.5 mIU/ml in the early follicular phase, 8.7–76.3 mIU/ml in the peri-ovulatory phase and 0.5–16.9 mIU/ml in the mid-luteal one with a detection limit of 0.07 mIU/ml. FSH ranges: 2.5–10.2 mIU/ml in the early follicular phase, 3.4–33.4 mIU/ml in the peri-ovulatory phase and 1.5–9.1 mIU/ml in the mid-luteal one with a detection limit of 0.3 mIU/ml. All was measured by Advia Centaur XT Immunoassay System analyzer (Siemens) which uses competitive (estradiol) or direct (progesterone, FSH, LH) immunoassay and for quantification of reaction uses Chemiluminescent Acridinium Ester technology. The hormone blood levels at the three time points of the MC are reported in Table 1.

### 2.3 Ultrasound evaluation

Transvaginal pelvic ultrasonography was performed in the early follicular phase. Scans were performed using a 4–10 MHz endocavitary transducer (GE Healthcare, Milwaukee, WI). Both the uterus and the ovaries were visualized, to address the presence of abnormal findings, such as endometrial polyps, myomas, ovarian cysts, or other adnexal masses. None of the women presented uterine or ovarian anomalies.

### 2.4 Psychological evaluation

The psychological assessment was performed at each of the three phases of the MC. To quantify the self-esteem level, the Rosenberg self-esteem scale (Prezza et al., 1997) was adopted. The Ryff’s test was administered to examine the six dimensions of well-being (autonomy, environmental mastery, personal growth, positive relations with others, purpose in life, and self-acceptance; Ruini et al., 2003). Finally, in addition to BAI (Beck and Steer, 1990) and BDI (Beck et al., 1996) tests administered at the first experimental session (as inclusion/exclusion criteria, see above), the tests were re-administered at each time point to exclude the appearance of depressive/anxious symptoms.

### 2.5 MEG acquisition, preprocessing and source reconstruction

MEG acquisition, preprocessing, source reconstruction and synchrony estimation have been performed according to our previous works (Liparoti et al., 2021; Cipriano et al., 2023b, 2024; Romano et al., 2023; Polverino et al., 2024). All the women were scanned in three time points that were sequential. The first scanning could change, that is to say that in some women the first scanning was performed during a T1, in other ones at T2 or at T3. So, most of them were scanned in the same cycle or, at most, during two successive cycles (Table 1).

In detail, data were acquired using a MEG system composed of 154 magnetometers SQUID (Superconductive Quantum Interference Device) and 9 reference sensors. The entire acquisition process took place in a magnetically shielded room (ATB, Biomag, ULM, Germany) to reduce external noise. A tracking system (Fastrak, Polhemus®) was used to define the position and orientation of the subject’s head. MEG signals were acquired with a sampling frequency of 1,024 Hz.

Two consecutive, resting state, closed eyes, 3.5 min-long recordings (of which the entire recording was used in preprocessing steps) were acquired. Electrocardiography (ECG) and electro-oculography (EOG) were also co-registered during the scan. The preprocessing and source-reconstruction was performed by filtering the MEG data in the 0.5–48 Hz range applying a 4th-order Butterworth IIR band-pass filter using the Fieldtrip toolbox in MATLAB (Oostenveld et al., 2011). After that, a Principal Component Analysis (PCA) was performed to orthogonalize signals with respect to the reference signals, reducing the environmental noise. An Independent Component Analysis (ICA; Barbati et al., 2004) was used to remove any ECG and EOG artifact.

After the co-registration of MEG data with MRI T1-weighted images, we extracted the time series of 116 ROI, according to the Automated Anatomical Labeling (AAL) atlas parcellation (Tzourio-Mazoyer et al., 2002). We exploited the volume conduction model introduced by Nolte (2003) and applied the Linearly Constrained Minimum Variance (Van Veen et al., 1997) beamformer algorithm implemented in the Fieldtrip toolbox (Oostenveld et al., 2011). The resulting time series were then band-pass filtered in the following frequency bands: delta (0.5–4 Hz), theta (4–8 Hz), alpha (8–13 Hz), beta (13–30 Hz), and gamma (30–48 Hz). Cerebellar ROIs were removed due to the poor reliability of the MEG signals in the posterior cranial fossa. These regions included the cerebellar lobules of both the cerebellar hemispheres (ROIs from 91 to 108 of the AAL) and the cerebellar vermis (ROIs from 109 to 116 of the AAL). Hence, 90 ROIs were retained for further analyses. Synchrony estimation between brain areas was estimated using the phase linearity measurement (PLM; Sorrentino et al., 2019), a phase-based metric unaffected by volume conduction (Baselice et al., 2019). PLM was estimated between each couple of regions obtaining a functional connectome per each of the two MEG scans (test and re-test, respectively). This was performed in each of the canonical frequency bands.

### 2.6 Fingerprint analysis

At each of the three time points of the MC, we performed two MEG recordings separated by ~2 min, hence calculating two FCs, named test and retest. Based on them (Figure 1), we estimated the brain-fingerprinting of each subject in the three different MC phases. We started by creating frequency-specific identifiability matrices (IM), using the Pearson’s correlation coefficient between the test and re-test FCs. The IM has subjects as rows and columns and encodes the information about the self-similarity and the similarity of each subject with the others. The test FC of each subject was correlated to the retest FCs of all the subjects within the same phase of the MC (including herself; Amico and Goñi, 2018). So, the resultant IM embodies, in the main diagonal, the information inherent to homo-similarity (I-self, the similarity between FCs of the same individual), while data about hetero-similarity (I-others, i.e., the similarity of that subject’s FC with the whole group of the same MC phase) are represented by the off-diagonal elements. Then, we extracted the differential identifiability (I-diff), a score that estimates the subject-specific fingerprint level of a specific brain dataset, by subtracting the I-others value from the I-self value (Amico and Goñi, 2018). Moreover, to define the probability of correctly identifying a specific individual, we calculated the success rate (SR) of subject recognition within a specific phase. The SR was computed on the number of times (expressed as percentage) that each subject showed an I-self higher than the I-others (i.e., how many times an individual was more similar to themselves than to another individual of the same phase).

Finally, we set out to measure how much each subject’s FC was similar to the mean of the other subject’s FCs in the previous phase of the MC by computing the “cycle-specific fingerprinting” score (I-cyclical, IC). Similarly to Sorrentino et al. (2021) we built two block identifiability matrices (hybrid identifiability matrices, IMs) by crossing, for each individual, the FC test and re-test, respectively (Figure 1). In this case, the Identifiability matrix was represented by a block matrix, where the number of blocks equals the number of groups (i.e., three in this work, one for each phase of the MC, Figure 1B). The within-phase group blocks (green and pink blocks in Figure 1B) represent the IM within a specific MC phase (in the figure T1 phase for the green block and T2 phase for the pink block). The between blocks elements (i.e., the two bicolor blocks) encode the similarity (or distance) between the test–retest connectomes of the women belonging to two different phases of the MC. The top right bicolor block contains the similarities between the connectomes from the test session of the T1 phase with the connectomes of the retest session of the T1 phase, while the bottom-left bicolor block contains the similarities between the connectomes of the women during the re-test session of the T2 phase with the connectomes of the test session of the T1 phase. The same analysis was performed comparing the connectomes of the T2 phase with the T3 phase and the connectome of the T3 phase with the ones of the T1 phase. Then, the correlation coefficients were averaged for each individual, obtaining subject-specific IC scores for each MC phase, that represent the similarity to the previous MC phase and as consequence, how much the FC of each individual changed across the MC.

### 2.7 Edges of interest for fingerprint

To estimate the edgewise reliability of individual connectomes across the test and the re-test recordings, we used the intra-class correlation coefficient (ICC; Koch, 2006), a measure that quantifies the similarity of the elements belonging to the same phase group. In our case, the higher the ICC values, the greater the stability of an edge over different recordings (Sorrentino et al., 2021). We hypothesized that, in a functional connectome, the edges with higher ICC were those that could contribute more to subject identifiability. So, we conducted a fingerprint analysis by sequentially adding them on the basis of their ICC values. We carried out this analysis by adding 100 edges at each iteration (and computed the SR values) starting from the highest ICC value to the lowest. A null model was built by adding the edges in random order, 100 times at each iteration, to validate our findings.

### 2.8 Cycle-specific fingerprinting and subjective mood state

Since the IC score describes the similarity of a subject to the subjects in a previous MC phase, it also entails information about how much a given subject’s FC is changed. Following this line of reasoning, we hypothesized that such a parameter may be related to the subjective mood state that can vary in function on theMC phase. Since our aim was to assess mood aspects that had no clinical significance (women with PMDD or PMS were excluded), we built a multilinear regression model to predict self-esteem, well-being and mood scores based on the IC scores. For well-being dimensions we built six different models in which one of the six dimensions was used as a dependent variable; the independent variables were unchanged. A model with the total well-being score was also performed.

### 2.9 Regional contribution

We evaluated the regional contribution of specific brain areas to identifiability and subjective mood state. Based on the ICC values in the alpha band, we studied the nodal strength of the most reliable edges, thus determining the regions of interest (ROIs) with the greatest influence on subjective mood state and identifiability. We performed the current analysis on the basis of the data extracted by the comparison of the clinical connectome of T1 and T2 phases. We included 1,200 edges (because of the best IC values, see above for details) and later we selected the most influential edges (i.e., above the 90% percentile).

### 2.10 Statistics

Statistical analysis was performed in MATLAB 2021b. Differences in hormonal fluctuation and psychological parameters were investigated through the ANOVA test. We analyzed all the comparisons among the I-self, I-others and I-diff values using permutation testing, where the labels of the two phases were randomly allocated 10,000 times. Thus, we obtained a null distribution of the randomly determined differences by computing the absolute value of the difference of the phase group averages at each iteration (Nichols and Holmes, 2002). The relationship between variables was studied with Pearson’s correlation and the results were corrected for multiple comparisons using the False Discovery Rate (FDR; Benjamini and Hochberg, 1995), setting the significance level at *p*-value <0.05. A multilinear regression model was performed to predict self-esteem, well-being and mood scores based on the IC scores and six other predictors: age, education and hormone blood levels (progesterone, estradiol, LH and FSH; Shen et al., 2017). Multicollinearity was assessed through the variance inflation factor (VIF; Snee, 1983; Belsley et al., 2004). The model was validated by using the leave-one-out cross validation (LOOCV) framework (Varoquaux et al., 2017). Furthermore, similarly to the edgewise identification, the multilinear analysis was performed in an iterative scenario where the IC score was calculated using different subset of edges, based on their ICC values. In particular, we calculated the IC by adding 100 edges at each iteration in descending order of stability (from the highest to lowest ICC). For the regional contribution of specific brain areas to identifiability and self-perception status, we studied the nodal strength of the most reliable edges, thus determining the ROIs with the greatest influence on both self-perception status and identifiability. We included 1,200 edges (i.e., the best IC values) and selected the most influential edges (i.e., above the 90% percentile).

## 3 Results

### 3.1 Demographic features

In Table 1 we report the clinical and laboratory characteristics of our study population. The significant differences in the profile of sex hormones between the MC phases were in line with the well-known hormonal trends over the MC. There were no significant differences in subjective clinical features across the MC. None of the women had undetectable progesterone in one of the three MC phases.

### 3.2 Connectome fingerprint

After False Discovery Rate (FDR) correction, no statistically significant difference in identifiability parameters (i.e., I-self, I-others, I-diff) between the three time points was found. Additionally, a linear mixed model, with age, education and phase of cycle set as fixed effects and “subject” as random effect did not show any difference in identifiability parameters values (in each canonical frequency band, set as dependent variables) according to age, education and, above all, cycle phase. This result demonstrates that, within the limit of our experimental design (which included a two-minute interval between test and retest recording) the I-self did not change significantly over MC.

### 3.3 Edge-based identifiability

**Figure 2:**
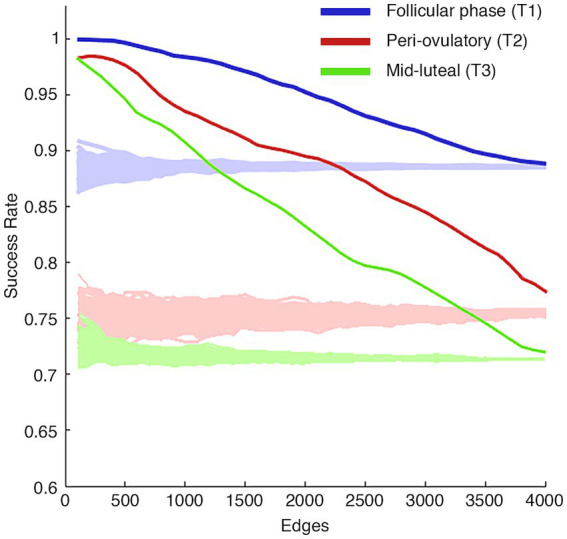
Iterative model of edgewise subject identification. The success rate (SR) distributions of T1, T2, and T3 phases, obtained by adding 100 edges at each step from the most to the least contributing ones, according to the intraclass correlation (ICC) values. The T1 phase (blue) quickly reached a complete SR (100%), and a slowly progressive decline but always preserving a great identifiability power. The T2 and T3 phases (red and green, respectively), equally to the T1, quickly reach an almost complete SR but start, after a few hundred edges, an important drop and a progressive loss in subject identification reaching a success rate of ~70–75% with 4,005 edges. The shaded areas of each figure represent the null distributions obtained by adding 100 edges at a time in a random order.

We used the one-way random-effects intra-class correlation coefficient (ICC) to test the edgewise reliability of the individual FCs. The edges with higher ICC values were those that contributed the most to the identifiability. We therefore investigated whether the identifiability of participants, at each MC phase, was dependent on the number of edges considered in the fingerprint analysis. We studied the distribution of success rate (SR) values (i.e., how many times, expressed as percentage, an individual was more similar to themselves than to another individual of the same phase) extracted from the fingerprint analysis by adding 100 edges per iteration, from the most to the least stable ones, based on ICC values. Our results showed different contributions to identifiability of the edges according to the MC phase. The women in the T1 phases quickly reached a complete SR (100%; Figure 2), maintaining a complete SR up to the 500 edges, where a slowly progressive decay in SR started without ever falling below 90%. The T2 and T3 phases also reached an almost complete SR (~98%) with a few ultrastable edges (right from 100 edges), but with a rapid and progressive decline in SR up to 4,005 edges (SR of ~70–75%).

### 3.4 Multilinear edge-based regression analysis and relationship with subjective mood state

We used the IC values to predict individual subjective mood states as assessed by the Rosenberg self-esteem scale, the Ryff’s test (for the six dimensions of well-being), the BAI and the BDI. We performed different edge-based multilinear regression models using the IC (alongside with age, education level and estradiol, FSH, LH, and progesterone hormone levels) as a predictor, and setting the subjective mood of interest as a dependent variable. The IC with the greatest predictive power was calculated, for each dependent variable, by adding 100 edges in descending order of stability at each iteration up to the full FC. Despite the participants’ age falling within a narrow range, we included it as a predictor to avoid the possibility that its influence on hormones could introduce bias in the prediction.

**Figure 3:**
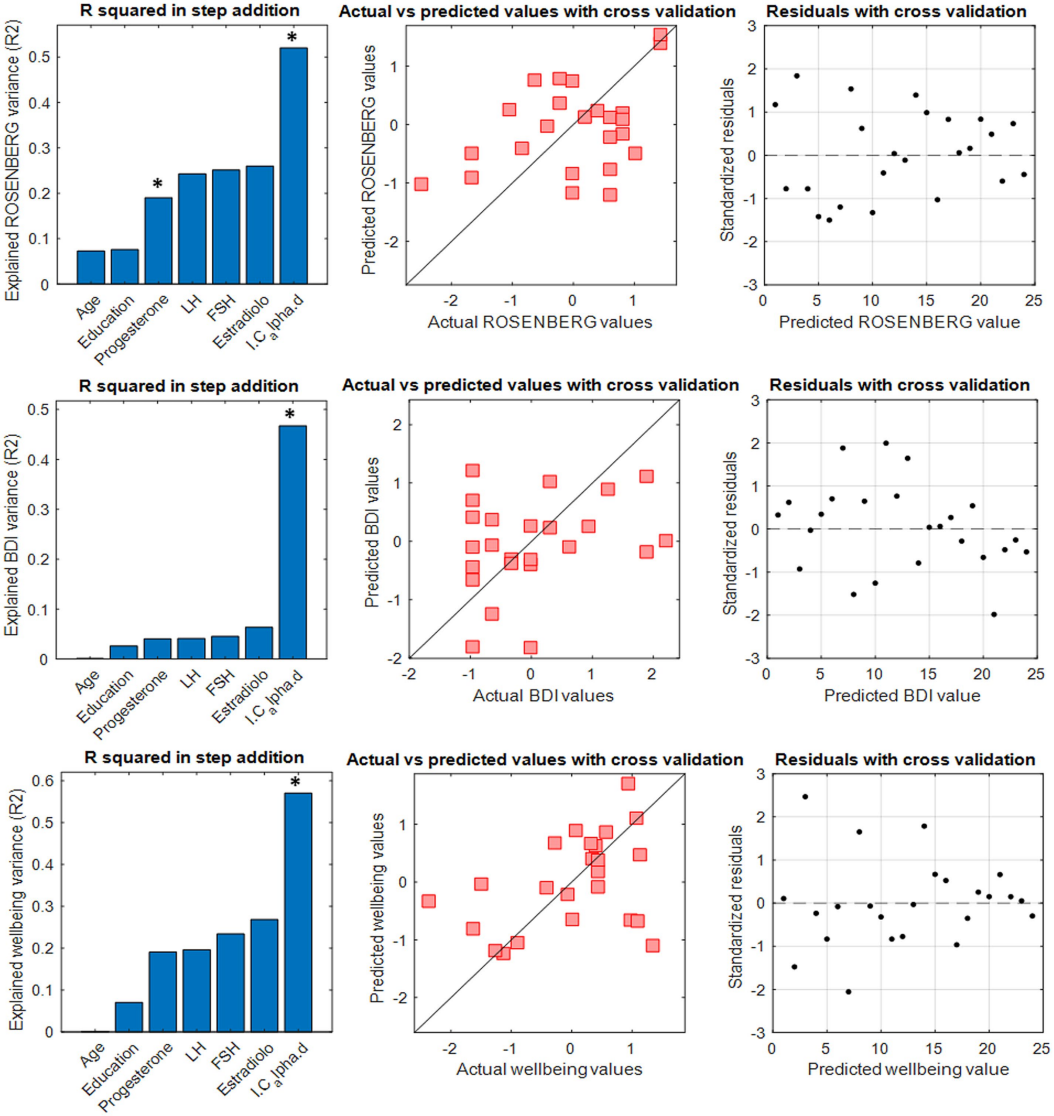
A multilinear regression model with leave-one-out cross validation (LOOCV) was performed to test the capacity of the *I-cyclical* to predict self-esteem, depression and well-being in the T2 phase. Panels on the left show the explained variance by the stepwise addition of the six predictors. The significant predictor is indicated with * in bold. The middle panels show the comparison between actual and predicted clinical features. Panels on the right show residuals distribution with cross-validation.

The IC in the alpha band significantly predicted the self-esteem level (*p*-value <0.01), mood (*p*-value <0.01) and five of the six the dimensions of well-being (*p*-value <0.01), save the autonomy. In nearly all the cases (Figure 3) the greatest predictive power was achieved when the IC was built with relatively few and highly stable edges (300 for BDI, 200 for Wellbeing and 1,200 for self-esteem). The IC did not show any significant predictive power over anxiety, as assessed by the BAI. The IC showed predictive power over the other parameters of the multilinear model including the hormonal ones that, with the only exception of the progesterone (inverse correlation) for the self-esteem prediction, did not show significant predictive power. To verify the independence of these results from the number of variables used in the model, linear models without the hormones (maintaining only the age and the IC as independent variables) were performed. The model maintained the high predictive power of the IC. The IC built in the other frequency bands did not show a comparable significant predictive power on mood and self-related aspects.

**Figure 4:**
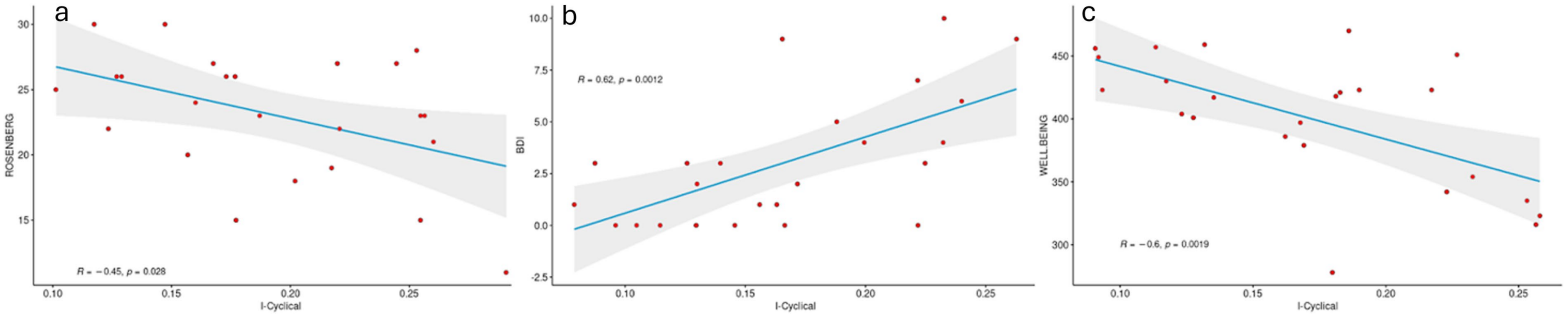
Pearson correlation between cycle-specific fingerprinting and subjective symptoms. The figure shows an inverse correlation between both self-esteem and wellbeing and IC and positive correlation between IC and depression. Higher scores at the Ryff and Rosenberg tests are related to positive outcomes, whereas higher BDI scores are indicative of worse outcomes.

Additionally, to evaluate the relationship between IC and self-esteem, mood and well-being, respectively, a Pearson’s correlation analysis, performed for each of these subjective conditions using the IC with greater predictive power, was carried out. A negative correlation was found between IC and both self-esteem (*r* = −0.45, *p*-value = 0.028, Figure 4A) and well-being (*r* = −0.6, *p*-value = 0.002, Figure 4C), whereas a positive correlation was observed with BDI (*r* = 0.62, *p*-value = 0.001, Figure 4C).

Finally, we performed the same analyses comparing the other phases of the MC to each other (i.e., T1-T3 and T3-T2) without finding statistically significant predictive power of IC in predicting the subjective state.

### 3.5 Regional contribution to identifiability and subjective mood state

**Figure 5:**
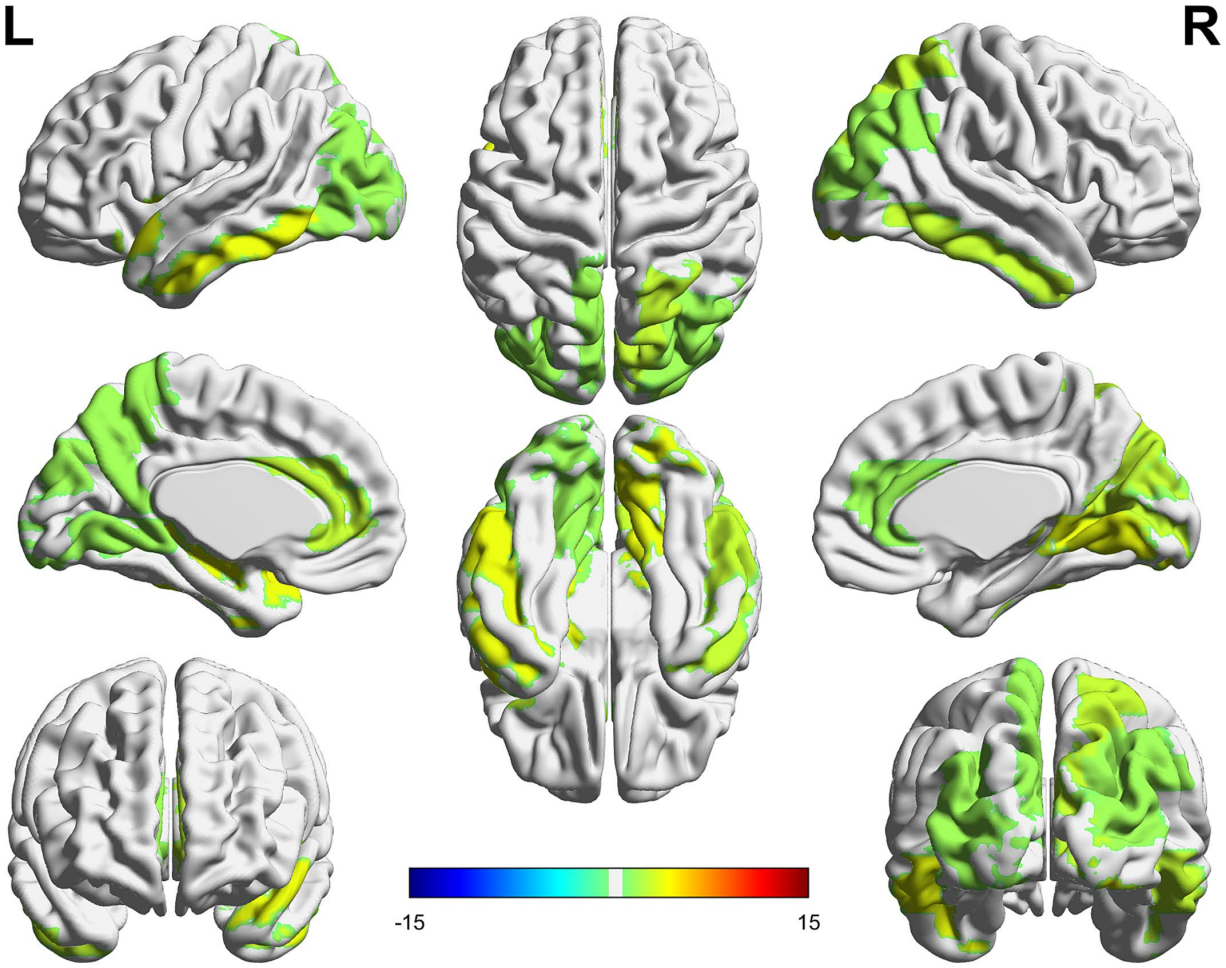
The colored areas represent the ROIs with the greatest nodal strength in identifiability and predicting subjective state. The nodal strength was calculated on the intraclass correlation matrices representing the stability between phases T1 and T2.

The ROIs with the greatest stability between T1 and T2 phases, hence contributing the most to the identifiability and in predicting the subjective outcome (in the T2 phase), were mainly located in the midline and posterior areas such as the anterior cingulate, the occipital (superior and middle), the calcarine, the lingual and the temporal inferior cortices on the left side and the superior parietal, the occipital (superior, middle and inferior), the lingual, the cuneus, the calcarine and the temporal middle cortices in the right hemisphere (Figure 5).

## 4 Discussion

The aim of the present work was to verify the stability, and therefore the reliability, of the brain fingerprint during the menstrual cycle. We subsequently verified this stability as a function of the number of FC connections necessary to achieve high identifiability and which brain areas play a major role in this aspect. Finally, given the variability of the women’s subjective mood state across the MC, the possible correlation between the latter and the brain fingerprint was investigated.

We firstly demonstrated that the FC fingerprinting is highly reliable and that, despite the effect that the MC has on the brain connectivity, the subject’s specific FCs remain identifiable regardless of the MC phase. Afterward, we investigated the contribution of specific edges to the identifiability by comparing the early follicular (T1), peri-ovulatory (T2) and mid-luteal (T3) phases. We found that, although the three phases (T1, T2 and T3) reached almost a complete SR (100%) with relatively few stable edges, the T2 and T3 MC phase identification dropped after only a few hundred edges, descending under 80% of the SR with the total number of edges. These data, along with the low SR of the T2 and T3 null models (when compared with the T1 null model), suggested a greater disparity of the edge stability in T2 and T3 phases. In view of this, the peri-ovulatory and mid-luteal phases showed a less stable (i.e., less ordered and more variable over time) brain connectome compared to the T1 phase. This feature was particularly evident in the anterior regions (being present a lower number of edges with great identifiability power in the anterior areas, since the more stable edges are located mainly in the posterior regions).

These results find partial confirmation in our recent study (Liparoti et al., 2024), where the peri-ovulatory phase was characterized by a more flexible dynamics as compared to the early-follicular phase. In this study, we used the technique based on the concept of “avalanche,” to investigate the variation of the size of the functional repertoire (i.e., brain’s flexibility) during the MC, by using source-reconstructed MEG data. We found that the neuronal avalanches underwent profound rearrangements especially during the peri-ovulatory phase, as compared to the early follicular phase. A flexible dynamics conveys the tendency of the brain to explore different configurations. Therefore, the greater brain flexibility that characterizes the peri-ovulatory phase may be the basis of the difference in edge stability between the T2 and T1 phases. A greater flexibility can be seen as the ability of a brain to reconfigure its pathways to maintain a proper functioning. So, the greater stability across the MC could represent a reduced ability of the brain to adapt to the physiological changes that happen during the different phases of the MC. In other words, higher edge stability might imply that the brain is less efficient in reconfiguring its neural circuits in response to the physiological variations associated with the MC, which could have implications for the mood state of women during these phases. These changes in brain dynamics during the MC phases have been also reported by previous fMRI studies that investigated the whole-brain dynamics during the MC showing that the hormonal changes can impact on brain dynamics across large-scale brain networks (Pritschet et al., 2020; De Filippi et al., 2021; Greenwell et al., 2023). In particular, in a fMRI study, specific brain areas such as prefrontal, limbic and subcortical regions exhibited a greater flexibility during the ovulatory phase as compared to the follicular and luteal ones (Mueller et al., 2021). Similar results were reported in a more recent article by Avila-Varela et al. that confirmed, in a fMRI protocol, the highest dynamical complexity (i.e., variability over time) in the preovulatory phase compared to the early follicular and mid-luteal phases (Avila-Varela et al., 2024). In addition, they found a direct modulation of estradiol and progesterone on the whole-brain, DMN, limbic, dorsal attention, somatomotor, and subcortical networks suggesting that ovarian hormones modulate brain network dynamics across the MC.

To verify whether these changes in edge stability between the T2 and T1 phases were associated with changes in subjective self-perception and mood, we built the I-cyclical (IC) on the basis of ICC and, after that, we conducted a multilinear regression model using the IC as a predictor variable. The IC in the alpha band was able to predict self-esteem, well-being and mood. This observation is in according with previous EEG and MEG studies which demonstrated that changes along the MC were mainly confined to the alpha band (Bazanova et al., 2014; Bröltzner et al., 2014), notoriously linked to the thalamic activity (Hughes and Crunelli, 2005). This is an intriguing aspect considering previous evidence of the ability of the ovaries to affect, directly and indirectly (through the cholinergic pathway of the Meynert nucleus), the physiological firing of the thalamus (Haraguchi et al., 2021). Noteworthy, the thalamic-modulated alpha rhythm propagation is mainly directed to the posterior brain areas (Halgren et al., 2019) which, interestingly, are the brain regions that in our study contributed more to prediction of self-esteem, wellbeing and mood. Hence, one might speculate that MC-related biochemical fluctuations might affect posterior brain activities via a regulation of the thalamic activities and that, in turn, these changes could affect the subjective self-perception and mood.

Concerning the alpha band and its association with these mood aspects, previous studies supported the central role of this frequency band in self-perception and well-being. A small number of studies investigated the neural correlates of wellbeing through EEG analyses and only few of them found significant relationships between single frequency bands and well-being (de Vries et al., 2023). A recent study with a large sample reported that the profile of high alpha and delta power and low beta was associated with higher well-being (Chilver et al., 2021). However, more than the alpha power, it seems to be relevant the alpha symmetry that was positively associated with well-being in several studies supporting the theory of the relationship between the alpha asymmetry and positive feelings (Harmon-Jones and Gable, 2018). Concerning self-esteem, previous studies have found that frontal alpha (as well as theta) band power decreased while participants experienced spontaneous self-referential thoughts (Bocharov et al., 2019) and that the frequency of self-referential thoughts during resting state was best predicted by higher alpha activity within the posterior brain networks (Knyazev et al., 2012). The central role of the alpha waves in the self-referential processing was also demonstrated by a recent study that investigated the sense of self through an EEG study. They found that somatic self-referential processing induced lower alpha in the frontal and insula cortex and higher alpha in the parietal cortex (Bao and Frewen, 2022). All these EEG studies seem to suggest that alpha rhythm plays an important role in self-referential processing and well-being.

It is also important to notice that we did not find (with the exclusion of the progesterone predictive power for the Rosenberg score) significant relationships between the blood levels of sex hormones and self-perception/brain connectivity parameters. Nevertheless, one should keep in mind that the biochemical changes across the MC are limited by no means to the fluctuations of the four biomarkers investigated in the present article but include a much wider set of metabolic and endocrine factors. The brain connectome is probably influenced by a complex interaction among all these factors. This could explain why the IC (that is itself the result of such complex biochemical interplay) benefits from a greater predictive power as compared to the levels of the hormones that we had sampled in this study. Further studies, including a more thorough investigation of the hormonal and metabolic profiles are warranted to clarify some of these aspects.

Concerning the brain connectivity correlates, the brain regions with the greatest contribution to self-esteem, wellbeing and mood prediction overlapped with the brain areas previously described as the areas highly influenced by sex hormone fluctuations, as well as with the most frequently reported by the studies that addressed the large-scale neural substrates of self-esteem, well-being and mood neural correlates (Dreher et al., 2007; Jung et al., 2014; Maller et al., 2014; Thimm et al., 2014; Teng et al., 2018; van Schie et al., 2018; Telzer et al., 2020; Chen et al., 2021). In fact, a follicular/luteal dependence of the activities of specific brain regions has been demonstrated in fMRI studies that investigated the relationship among sex hormones, ROI-specific brain activity and cognitive and mood aspects (see Catenaccio et al., 2016; Dubol et al., 2021). The cingulate cortex was the most commonly influenced by the hormonal fluctuations (Dreher et al., 2007; Thimm et al., 2014) followed by the middle and inferior temporal, lingual and fusiform gyri and parietal and occipital cortex (Dreher et al., 2007; Thimm et al., 2014; Pletzer et al., 2019).

Additionally, it has been previously reported the association between the posterior and midline brain regions and well-being, self-esteem and mood highlighting the importance of these areas in self-concept and self-referential aspects, as well as in mentalization and motivation circuits (Yang et al., 2016; van Schie et al., 2018; Telzer et al., 2020). The midline structures have been suggested to be involved in the self-referential aspects by encoding self-relatedness of stimuli and by regulating self-related negative and positive stimuli (van Schie et al., 2018; Telzer et al., 2020) whereas the temporal lobe contributes to the self-referential processing and autobiographical memory promoting a memory-based construction of the self (Andrews-Hanna, 2012; Yang et al., 2016). The posterior brain regions, in particular the lingual gyrus, the cuneus and the middle occipital gyrus are involved in positive social feedback (Eisenberger et al., 2011; Pan et al., 2016; Yang et al., 2016), contributing to self-referential processing and social cognition (Kircher et al., 2000; D’Argembeau et al., 2007; Brühl et al., 2014), and their activation is associated with negative self-appraisal (Brühl et al., 2014), self-criticism (Longe et al., 2010) and the retrieval of self-encoded negative personality traits (Fossati et al., 2004).

In the present work we also investigated the trend of the relationship between IC in the alpha band and subjective psychological assessment. We found a negative relationship between both self-esteem and wellbeing and IC, along with a positive correlation between IC and depression. Higher scores at the Ryff and Rosenberg tests are related to positive outcomes, whereas higher BDI scores are indicative of worse outcomes. This means that the higher the stability of the connections that involve posterior regions across the MC phases, the worse the outcomes (lower self-esteem and wellbeing as well as increased risk of depression).

In summary, in the present study we demonstrate that during the MC the brain fingerprint maintains a high stability which in the peri-ovulatory and mid-luteal phase is slightly lower than in the early follicular phase which however does not affect its reliability. Moreover, the reduced stability of the functional connections of the posterior areas was associated with higher well-being and self-esteem, as well as with a better mood.

However, the current study is not exempt from some limits. Firstly, the small sample size probably represents the main limit of the study. Furthermore, according to the experimental design of the study, the women included are a selected population. Previous pregnancies, breastfeeding periods, the use of oral contraceptives as well as premenstrual symptoms can frequently characterize the life of a woman; for this reason, the choice of a sample without these features reduces the generalizability of these findings. Another limit of the study could be represented by the self-esteem assessment. In fact, the Rosenberg scale evaluates this aspect in a global manner without exploring in detail the various traits of self-esteem and their sub-components. Finally, we did not find (with the exclusion of the progesterone predictive power for the Rosenberg score) significant relationship between sex hormone blood levels and emotional/brain connectivity parameters. However, it is important to recall that the biochemical changes across the MC are not only about the fluctuation of the four biomarkers investigated in the present article, but include a wider group of other metabolic and endocrine factors. The brain connectome is probably influenced by a complex interaction among all these factors. This could explain why IC (that is itself the result of this complex biochemical interplay) benefits from a greater predictive power as compared to a single hormone. So, further studies, including a larger cohort, less stringent exclusion criteria and a more thorough investigation of the hormonal and metabolic profiles as well as more specific psychometric tools are necessary to confirm these results. Concerning some aspects of the ROIs selection, we should remember that AAL is a structural atlas, and as such, could represent a limitation for studies on the brain connectivity. These atlases typically rely on macro-anatomical boundaries that may not align with the both functional or microstructural organization of the brain, risking a misrepresentation of functionally relevant networks. However they remain a valuable tool for studying brain connectivity because they provide a standardized framework for dividing the brain into regions of interest, enabling consistent and reproducible assessment of connectivity across studies. In absence of cerebellar ROIs, we cannot conclude whether the cerebellum is involved or not in the MC-related brain connectivity changes. This represents a limitation, primary due to the intrinsic characteristics of the MEG, and could be better investigated in the future by integrating high temporal resolution techniques (e.g., MEG or EEG) with MRI which provides a better resolution of the cerebellar regions. Also, we found high predictive power on the self-perception sphere only in the peri-ovulatory phase but not in the early-follicular and the mid-luteal phases. This aspect may be due to a less marked difference between T2-T3 and T3-T1 in edge stability that might not be observable given the small size of our cohort. This aspect seems to be supported by a recent study on brain connectivity reconfiguration patterns across the MC (Liparoti et al., 2024) that found a clear difference in the stability of the brain dynamics only in T2-T1 comparison.

## Data Availability

The raw data supporting the conclusions of this article will be made available by the authors, without undue reservation, upon explicit request to the corresponding author GS.
